# Research on Stacked Piezoelectric Cymbal Vibrator

**DOI:** 10.3390/mi14112039

**Published:** 2023-10-31

**Authors:** Xinhu Liu, Yajun Zheng, Yanming Guo, Ningdong Hu, Hongping Hu

**Affiliations:** 1 Department of Mechanics, School of Aerospace Engineering, Huazhong University of Science and Technology, Wuhan 430074, China; m202171652@hust.edu.cn (X.L.); m202271679@hust.edu.cn (Y.G.); huningdong2011@163.com (N.H.); 2 Hubei Key Laboratory for Engineering Structural Analysis and Safety Assessment, Huazhong University of Science and Technology, Wuhan 430074, China; 3 Shanghai Ruisheng Kaitai Acoustic Science and Technology Co., Ltd., Shanghai 201100, China; zheng.yj@outlook.com

**Keywords:** cymbal vibrator, piezoelectric stack, transfer-matrix method, finite element method, displacement amplification

## Abstract

As demand for haptic feedback increases, piezoelectric materials have become one of the best candidate materials due to their small size, high electromechanical coupling coefficient, and fast response. A stacked piezoelectric cymbal vibrator is proposed based on the common cymbal-type transducer, which is composed of a piezoelectric stack to drive and a cymbal disk to amplify displacement. A coupling theoretical model between the piezoelectric stack and the cymbal-type structure is established. The longitudinal and radial displacements of the stacked piezoelectric cymbal vibrator are calculated in the low frequency range (<1000 Hz) by the theoretical model and the finite element method. The theoretical and numerical results are in good agreement. The results show that the radial displacement can be converted into longitudinal displacement and then effectively amplified by the cymbal disk with an amplification ratio of 30. The feature is conducive to its widespread application in the field of consumer electronics.

## 1. Introduction

With the advancement of science and technology, the information and internet industries have developed rapidly. In recent years, technologies such as smart wearable devices and virtual reality have begun to enter people’s lives, and people’s demand for bionic tactile feedback is increasing. Many companies have also shifted their focus to haptic feedback [1,2,3,4]. At present, most of the vibration motors in consumer electronic products are realized by electromagnetic driving, such as the eccentric vibrating motor and linear vibrating motors commonly used in smart phones. These motors can simulate press feedback by electromagnetic vibration, which can achieve a similar effect without mechanical structure, and improve the reliability of the structure while enhancing the waterproof property of the structure [5,6,7,8]. However, electromagnetic motors are prone to electromagnetic interference and can only work within the natural frequency range [9]. In contrast, piezoelectric vibration motors do not produce electromagnetic interference, have a wider working frequency, and have a richer vibration effect [10,11,12]. However, because of their small size, piezoelectric materials make it difficult to produce large displacements and accelerations, so the vibration is weak [13,14,15,16]. The cymbal transducer is a type of transducer composed of two cymbal-shaped structures and a piezoelectric ceramic, which has the advantages of a simple structure, small size, and wide frequency band. At the same time, due to the amplification of the displacement by the cymbals, the whole structure has a high electromechanical coupling coefficient [17,18]. Traditional cymbal transducers are usually made of single-layer piezoelectric ceramics [17], but single-layer ceramic has a low electromechanical coupling coefficient, and the displacement or generated voltage is relatively small even with an amplification structure. Piezoelectric stacks have the advantages of precise control, sensitive response, and large output force; hence, they have wide applications in semiconductor technology, aerospace, image processing, astronomy, and other fields [19,20,21,22,23]. Piezoelectric stacks are made by sintering multiple layers of ceramics into a layered structure, which is further electrically connected in parallel or series between the layers. Under the same voltage or force, piezoelectric stacks can generate greater displacement or a higher voltage than those with a single layer. Therefore, stacked structures are often chosen as structures for piezoelectric devices.

In previous studies, cymbal transducers were often used as sensors and were widely applied in underwater communication, sonar, micro-electromechanical, and medical fields [18,24,25,26,27,28,29]. Many scholars have conducted corresponding research on cymbal transducers. After analyses and optimization, cymbal transducers were achieved with a good equivalent piezoelectric coefficient and power generation effect [30,31,32,33,34,35]. However, most research was mainly conducted through experiments or finite element simulations [17,31,36,37,38]. Cymbal transducers lack theoretical analysis, especially without considering the coupling between piezoelectric stacks and cymbals [39,40,41,42]. The vibration characteristics of cymbal transducers are still not revealed.

In this paper, we propose a novel cymbal vibrator suitable for tactile feedback vibration, which uses a piezoelectric stack to drive two cymbal disks to vibrate, effectively increasing the vibration displacement. Firstly, the vibration characteristics of piezoelectric stacks and cymbal disks are studied, respectively, under given boundary conditions. Then, by setting continuity coupling conditions, a voltage-displacement theoretical model of the whole structure is established, which agrees well with the simulation result below 1000 Hz.

## 2. Theoretical and Simulation Analysis of Piezoelectric Stack

**Figure 1:**
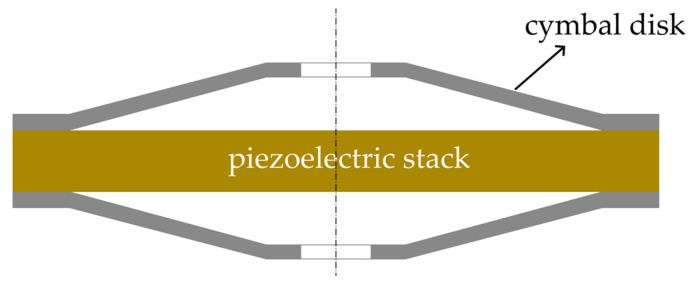
Cross section of the cymbal vibrator.

As shown in Figure 1, the stacked cymbal vibrator is composed of a piezoelectric stack and two cymbal disks. The circular piezoelectric stack is installed in the middle of the cymbal vibrator. The lower edge of the cymbal disk is bonded to the stack, while a small hole is introduced at the top of the cymbal disk to maintain air pressure balance and reduce noise [43].

**Figure 2:**
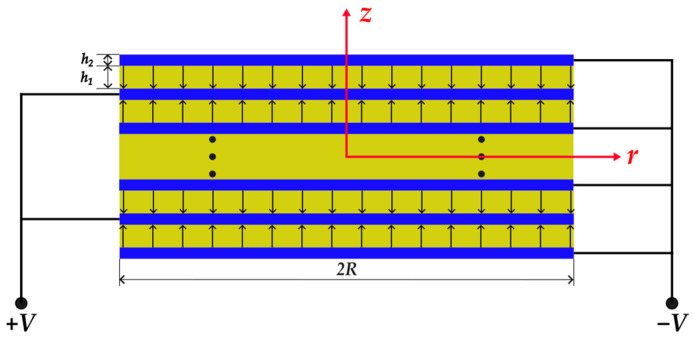
Cross section of the piezoelectric stack.

As shown in Figure 2, the circular piezoelectric stack consists of piezoelectric layers colored yellow and electrode layers colored blue, alternately. The polarization directions of adjacent piezoelectric layers are opposite, as shown by the arrows. The upward polarization direction is along the positive *z*-axis. The thicknesses of each electrode layer and each piezoelectric layer are denoted by *h*_1_ and *h*_2_, respectively. The diameter of the stack actuator is represented by 2*R*. PZT-5H is chosen as the piezoelectric material. Silver is chosen as the electrode material for the piezoelectric stack due to its good conductivity. The major material parameters are listed in Table 1. Other material parameters are also cited in reference [44].

### 2.1. Theoretical Analysis of Piezoelectric Stack

The linear constitutive relation of piezoelectric materials can be described by the second type of piezoelectric equation [44].

(1) $$\begin{array}{l} T_{i}=c_{ij}^{E}S_{j}-e_{ki}E_{k}\\ D_{m}=e_{mj}S_{j}+\varepsilon_{mk}^{S}E_{k} \end{array}$$

where *T_i_* and *S_j_* are the stress and strain tensors with *i*, *j* = 1, 2, 3, …, 6, and *D_m_* and *E_k_* are the electric displacement and electric field vectors with *m*, *k* = 1, 2, 3. $c_{ij}^{E}$ are the stiffnesses under a constant electric field, *e_ki_* are the piezoelectric stress constants, $\varepsilon_{mk}^{S}$ and are the clamped dielectric constants.

To the cylindrical coordinate system, (r, θ, z) corresponds to (1, 2, 3) so that the positive poling direction corresponds to 3. The motion of an axisymmetric stack satisfies

(2) $$u_{\theta }=0,\;\frac{\partial }{\partial \theta }=0$$

Therefore, the displacement of the disk can be expressed by the radial displacement $u_{1}\left(r,z,t\right)$ and the longitudinal displacement $w_{1}\left(r,z,t\right)$, where t is the time. The normal strain $S_{r}{}_{r}$, $S_{\theta }{}_{\theta }$, $S_{zz}$, and the shear strain $S_{zr}$ can be expressed by the following geometric equations.

(3) $$S_{r}{}_{r}=\frac{\partial u_{1}}{\partial r},\;S_{\theta }{}_{\theta }=\frac{u_{1}}{r},\;S_{zz}=\frac{\partial w_{1}}{\partial z}$$

(4) $$2S_{zr}=\frac{\partial u_{1}}{\partial z}+\frac{\partial w_{1}}{\partial r}$$

In the piezoelectric layer, the relationship between the electric field $E_{z}$ and the electric potential $\phi \left(z,t\right)$ is given by

(5) $$E_{z}=-\frac{\partial \phi }{\partial z}$$

The electrical displacement satisfies the Gauss theorem.

(6) $$D_{i,i}=0$$

The piezoelectric layer also satisfies the equilibrium differential equation.

(7) $$\begin{array}{l} \frac{\partial T_{rr}}{\partial r}+\frac{T_{rr}-T_{\theta \theta }}{r}+\frac{\partial T_{zr}}{\partial z}=\rho_{1}\frac{\partial^{2}u_{1}}{\partial t^{2}}\\ \frac{\partial T_{zr}}{\partial r}+\frac{T_{zr}}{r}+\frac{\partial T_{zz}}{\partial z}=\rho_{1}\frac{\partial^{2}w_{1}}{\partial t^{2}} \end{array}$$

Substituting the geometric Equations (3) and (4) into the constitutive Equation (1), the expressions of stresses $T_{ij}$ and electric displacement $D_{z}$ are obtained.

(8) $$\begin{array}{l} T_{rr}=c_{11}^{E}\frac{\partial u_{1}}{\partial r}+c_{12}^{E}\frac{u_{1}}{r}+c_{13}^{E}\frac{\partial w_{1}}{\partial z}+e_{31}\frac{\partial \phi }{\partial z}\\ T_{\theta \theta }=c_{12}^{E}\frac{\partial u_{1}}{\partial r}+c_{11}^{E}\frac{u_{1}}{r}+c_{13}^{E}\frac{\partial w_{1}}{\partial z}+e_{31}\frac{\partial \phi }{\partial z}\\ T_{zz}=c_{13}^{E}\frac{\partial u_{1}}{\partial r}+c_{13}^{E}\frac{u_{1}}{r}+c_{33}^{E}\frac{\partial w_{1}}{\partial z}+e_{33}\frac{\partial \phi }{\partial z}\\ T_{zr}=c_{44}^{E}\left(\frac{\partial u_{1}}{\partial z}+\frac{\partial w_{1}}{\partial r}\right)\\ D_{z}=e_{31}\frac{\partial u_{1}}{\partial r}+e_{31}\frac{u_{1}}{r}+e_{33}\frac{\partial w_{1}}{\partial z}-\varepsilon_{33}\frac{\partial \phi }{\partial z} \end{array}$$

For the thin plate, the normal stress in the thickness direction cannot depart much from zero. Thus, it is assumed to vanish throughout $T_{zz}=0$. From the third formula in Equation (8), we obtain

(9) $$\frac{\partial w_{1}}{\partial z}=-\frac{c_{13}^{E}}{c_{33}^{E}}\frac{\partial u_{1}}{\partial r}-\frac{c_{13}^{E}}{c_{33}^{E}}\frac{u_{1}}{r}-\frac{e_{33}}{c_{33}^{E}}\frac{\partial \phi }{\partial z}$$

Then, the Equation (8) can be rewritten as

(10) $$\begin{array}{l} T_{rr}=c_{11}^{p}\frac{\partial u_{1}}{\partial r}+c_{12}^{p}\frac{u_{1}}{r}+e_{31}^{p}\frac{\partial \phi }{\partial z}\\ T_{\theta \theta }=c_{12}^{p}\frac{\partial u_{1}}{\partial r}+c_{11}^{p}\frac{u_{1}}{r}+e_{31}^{p}\frac{\partial \phi }{\partial z}\\ T_{zr}=c_{44}^{E}\left(\frac{\partial u_{1}}{\partial z}+\frac{\partial w_{1}}{\partial r}\right)\\ D_{z}=e_{31}^{p}\frac{\partial u_{1}}{\partial r}+e_{31}^{p}\frac{u_{1}}{r}-\varepsilon_{33}^{p}\frac{\partial \phi }{\partial z} \end{array}$$

where

(11) $$\begin{array}{l} c_{11}^{p}=c_{11}^{E}-{\left(c_{13}^{E}\right)}^{2}/c_{33}^{E},\;c_{12}^{p}=c_{12}^{E}-{\left(c_{13}^{E}\right)}^{2}/c_{33}^{E}\\ e_{31}^{p}=e_{31}-c_{13}^{E}e_{33}/c_{33}^{E},\;\varepsilon_{33}^{p}=\varepsilon_{33}^{S}-e_{33}/c_{33}^{E} \end{array}$$

Substituting Equation (10) into Equation (7) yields

(12) $$c_{11}^{p}\left(\frac{\partial^{2}u_{1}}{\partial r^{2}}+\frac{1}{r}\frac{\partial u_{1}}{\partial r}-\frac{u_{1}}{r^{2}}\right)=\rho_{1}\frac{\partial^{2}u_{1}}{\partial t^{2}}$$

For a steady-state problem with an angular frequency ω, it becomes

(13) $$\frac{\partial^{2}u_{1}}{\partial r^{2}}+\frac{1}{r}\frac{\partial u_{1}}{\partial r}\left(\xi_{1}^{2}-\frac{1}{r^{2}}\right)u_{1}=0$$

where $\xi_{1}=\sqrt{\omega^{2}\rho_{1}/c_{11}^{p}}$. Then, the general solution of $u_{1}$ is

(14) $$u_{1}=BJ_{1}(\xi_{1}r)e^{i\omega t}$$

where *B* is an integration constant. i is an imaginary unit. $J_{1}$ is the first Bessel function of the first order.

As an isotropic material, the silver electrode is then studied. Its physical parameters are distinguished by subscript 2. Its stresses can also be obtained [45,46].

(15) $$\begin{array}{l} \sigma_{r2}=\lambda \left(\frac{\partial u_{2}}{\partial r}+\frac{u_{2}}{r}+\frac{\partial w_{2}}{\partial z}\right)+2G\frac{\partial u_{2}}{\partial r}\\ \sigma_{\theta 2}=\lambda \left(\frac{\partial u_{2}}{\partial r}+\frac{u_{2}}{r}+\frac{\partial w_{2}}{\partial z}\right)+2G\frac{u_{2}}{r}\\ \sigma_{z2}=\lambda \left(\frac{\partial u_{2}}{\partial r}+\frac{u_{2}}{r}+\frac{\partial w_{2}}{\partial z}\right)+2G\frac{\partial w_{2}}{\partial z}\\ \tau_{zr2}=G\left(\frac{\partial u_{2}}{\partial z}+\frac{\partial w_{2}}{\partial r}\right) \end{array}$$

where $u_{2}$ and $w_{2}$ are the displacements in radial and thickness directions of the electrodes, respectively, λ and *G* are Lamé constants, and *G* is also the shear modulus of the electrodes, which can be expressed in terms of Young’s modulus and Poisson’s ratio.

(16) $$\lambda =\frac{\mu_{2}E_{2}}{\left(1+\mu_{2}\right)\left(1-2\mu_{2}\right)},\;G=\frac{E_{2}}{2\left(1+\mu_{2}\right)}$$

Similarly, the normal stress of the electrode in the thickness direction is assumed $\sigma_{z2}=0$ to be from Equation (15), we have

(17) $$\frac{\partial w_{2}}{\partial z}=-\frac{\lambda }{\lambda +2G}\left(\frac{\partial u_{2}}{\partial r}+\frac{u_{2}}{r}\right)$$

Then, Equation (15) can be rewritten as

(18) $$\begin{array}{l} \sigma_{r2}=\lambda_{1}^{p}\frac{\partial u_{2}}{\partial r}+\lambda_{2}^{p}\frac{u_{2}}{r}\\ \sigma_{\theta 2}=\lambda_{2}^{p}\frac{\partial u_{2}}{\partial r}+\lambda_{1}^{p}\frac{u_{2}}{r}\\ \tau_{zr2}=G\frac{\partial u_{2}}{\partial z} \end{array}$$

where

(19) $$\begin{array}{l} \lambda_{1}^{p}=\lambda +2G-\frac{\lambda^{2}}{\lambda +2G}\\ \lambda_{2}^{p}=\frac{2G\lambda }{\lambda +2G} \end{array}$$

The expression of $u_{2}$ can be obtained as

(20) $$u_{2}=AJ_{1}(\xi_{2}r)e^{i\omega t}$$

where $\xi_{2}=\sqrt{\omega^{2}\rho_{2}/\lambda_{1}^{p}}$. *A* is an integration constant.

For the multilayer piezoelectric stack, the radial displacements at the interface between the piezoelectric layer and the electrode layer satisfy the continuity condition as

(21) $$u_{1}=u_{2}$$

Boundary condition at the cylindrical surface of r=R, the radial resultant force satisfies

(22) $$n\left(\int_{0}^{h_{1}}T_{r}{}_{r}dz+\int_{h_{1}}^{h_{2}}\sigma_{r2}dz\right)=0$$

where *n* is the layer number of the piezoelectric layers. From Equations (3), (9), and (17), the strains *S_zz_* and $\varepsilon_{zz}$ can be expressed by their respective radial displacements. Then, the displacement $w_{z}$ in the thickness direction can be further calculated by

(23) $$w_{z}=nh_{1}S_{zz}+\left(n+1\right)h_{2}\varepsilon_{zz}$$

### 2.2. Analyses of the Piezoelectric Stack

**Figure 3:**
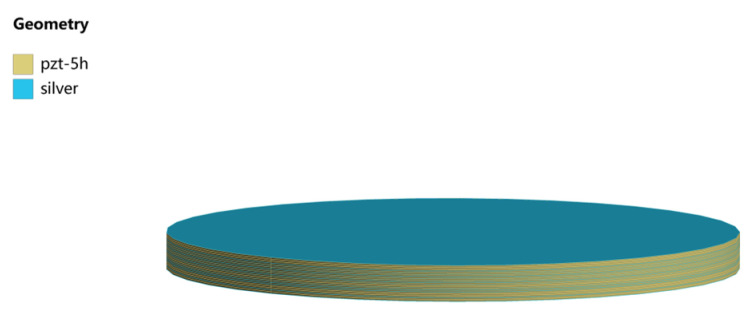
The piezoelectric stack model in Ansys Workbench.

As shown in Figure 3, the structure of the circular stack is composed of 19 piezoelectric layers and 20 electrode layers. The stack diameter is *2R* = 15 mm, the piezoelectric layer thickness is *h*_1_ = 45 μm, the electrode layer thickness is *h*_2_ = 5 μm, and the total thickness is 955 μm. The structure is modeled in Solidworks 2020 software. The piezoelectric layer and the electrode layer are connected by binding. The model is then imported into the ANSYS Workbench for calculation, where the design modeler is applied to preprocess the structure. For the multilayer piezoelectric stack, material properties are assigned in the ACT plug-in. Alternating voltages applied to electrode layers are +*V* and −*V*, as shown in Figure 2. For the boundary condition on the bottom, displacements in the thickness direction of all nodes are set to 0; moreover, for nodes on their circular edges, circumferential displacements are also set to 0. Hexahedral mesh is used, and the element size is 0.5 mm, and the results have good convergence under this element size.

#### 2.2.1. Static Analysis

Static analysis is performed on the Workbench. A voltage difference of 80 V is applied to the piezoelectric layers.

**Figure 4:**
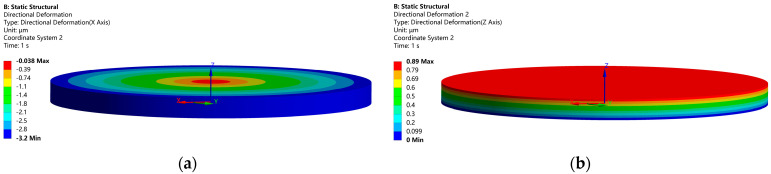
Displacement distribution of the structure from static analysis. (**a**) radial, (**b**) longitudinal.

**Figure 5:**
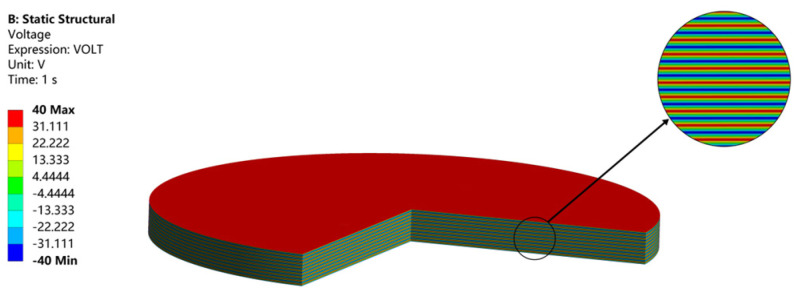
Electric potential distribution of the piezoelectric stack.

Figure 4 shows the displacements of the structure in the radial direction with a maximum value of 3.2 μm in (a); while the displacement in the thickness direction has a maximum value of 0.89 μm in (b). It can be noted that the radial displacement is one order of magnitude greater than the longitudinal displacement. If the radial displacement is transformed and amplified into a longitudinal displacement, a larger longitudinal displacement can be obtained. The electric potential distribution of the piezoelectric stack is shown in Figure 5. Each piezoelectric layer has an electric potential gradient and is therefore subjected to an electric field.

#### 2.2.2. Harmonic Response Analysis

**Figure 6:**
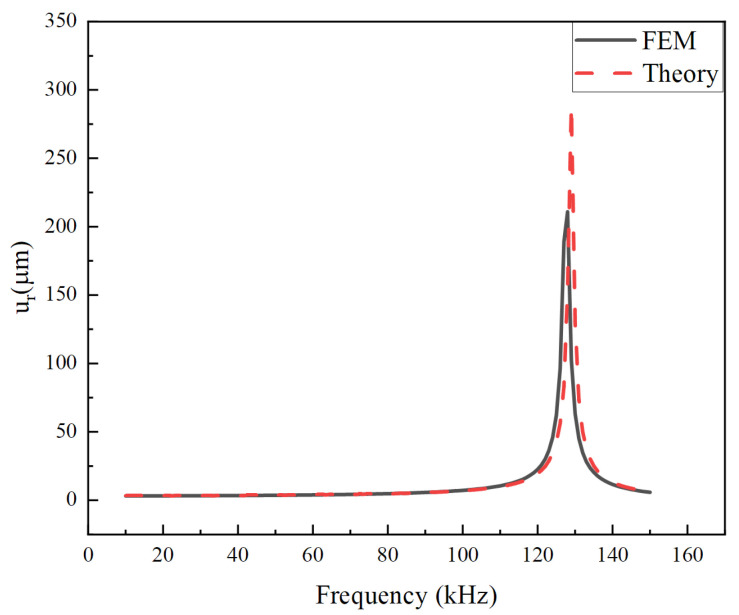
Radial displacement versus driving frequency is calculated by the finite element method (FEM) and theoretical model.

**Figure 7:**
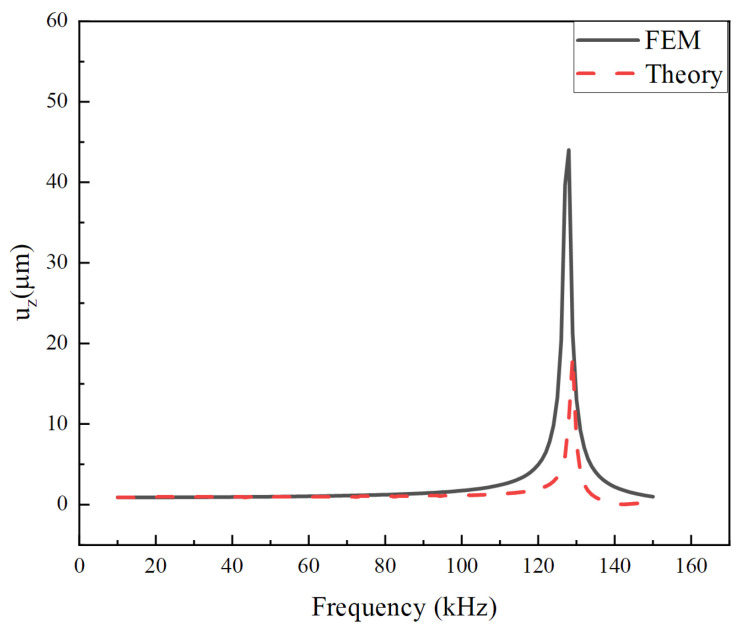
Longitudinal displacement versus driving frequency calculated by FEM and theoretical solution.

The harmonic analysis is performed by using the harmonic response module in Workbench with the full method. The global damping is set to 0.01. The boundary conditions are the same as for the static analysis. A sinusoidal voltage is applied in a certain frequency range. Figure 6 shows the radial displacement response of the stack in the frequency range from 10 kHz to 150 kHz under an 80 V voltage, calculated by theory and simulation, respectively. In order to correspond to the finite element calculation, the same material damping *η* = 0.01 is taken in the theoretical calculation [44]. As can be seen from the figure, the theoretical results agree well with the simulation values in the resonance region. The structure exhibits a resonance frequency of around 130 kHz. Figure 7 shows the comparison results of the longitudinal displacement under the same conditions. The resonance frequencies predicted by the two methods are very close, which mutually verifies the correctness of the calculation results. However, amplitudes do not match well at the resonant frequency. The phenomenon mainly stems from the difference between these two models. The theoretical model is an axisymmetric model that meets Equation (2). Therefore, the circumferential displacement is assumed to be 0. However, the finite element model is a three-dimensional model with circumferential displacement not equal to 0. Therefore, circumferential displacement affects radial displacement and thickness direction displacement, especially around the fundamental frequency of the piezoelectric stack. Fortunately, the operating frequency is much lower than the fundamental frequency.

## 3. Theoretical Model and Simulation of the Cymbal Disk

In the cymbal vibrator, when a voltage is applied to the piezoelectric element, the element not only produces longitudinal displacement but also contracts radially. The upper and lower cymbal disks can amplify the lateral shrinkage displacement and transfer it to the longitudinal direction by using the triangular amplification principle, which greatly increases the longitudinal displacement [47]. Then, the overall structure can obtain a high electromechanical coupling coefficient. In previous studies, the cymbal plate was usually regarded as a disc-shaped membrane spring and was calculated using the Almen–Laszlo formula [40]. The limitation of the method is that the displacement amplification ratio cannot be obtained. It is because the relationship between longitudinal force and longitudinal displacement is only considered; the radial force and deformation at the bottom are neglected by simplification [48]. Therefore, at first, we take all forces and displacements into account for the cymbal disk, then investigate the coupling between the piezoelectric stack and the cymbal disk.

### 3.1. Theoretical Model of the Cymbal Disk

**Figure 8:**
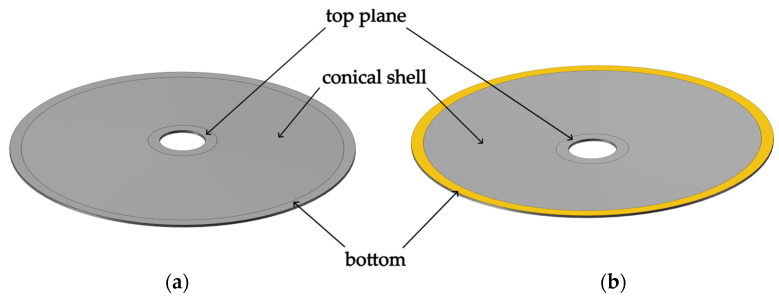
Cymbal disk. (**a**) top view, (**b**)bottom view.

**Figure 9:**
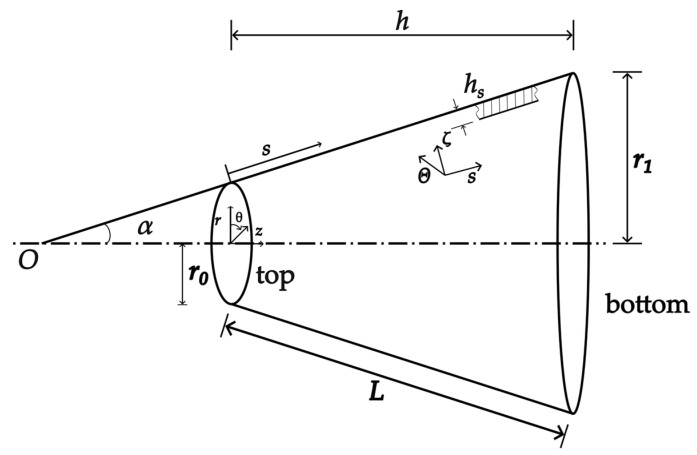
Conical shell model of the cymbal disk and coordinate systems.

As shown in Figure 8, the cymbal disk consists of a top plane, a conical shell, and a bottom. The cymbal disk has a small hole at the top. The air fills the cavity formed by the cymbal disk and piezoelectric ceramics. But due to the presence of the hole, we neglect the effect of air pressure. Because the displacement amplification effect is generated by the conical shell, while the other two parts have little impact on the amplification, the investigation is focused on the conical shell. The conical shell model and coordinate systems are shown in Figure 9. Unless otherwise stated, the material and specific dimensions are listed in Table 2.

As an axisymmetric conical shell model, its circumferential displacement *v* and derivative with respect to the circumference can be considered as

(24) $$v=0,\;\frac{\partial }{\partial \Theta }=0$$

Then, the governing equations of the conical shell are simplified as [49]

(25) $$\begin{array}{l} \frac{1}{s}\frac{\partial \left(sN_{s}\right)}{\partial s}-\frac{N_{\Theta }}{s}+\rho h\omega^{2}u=0\\ -\frac{1}{s\tan\alpha }N_{\Theta }+\frac{1}{s}\frac{\partial \left(sQ_{s}\right)}{\partial s}+\rho h\omega^{2}w=0 \end{array}$$

where *u* and *w* are displacements in *s* and ς directions, $N_{s}$, $N_{\Theta }$, $M_{s}$, $M_{\Theta }$, and $Q_{s}$ are the normal forces, bending moments, and shear forces under the corresponding coordinate axes, respectively. These forces and moments can be described as

(26) $$\begin{array}{l} N_{s}=D\left[\frac{\partial u}{\partial s}+\frac{\mu }{s}\left(u+\frac{w}{\tan\alpha }\right)\right]\\ N_{\Theta }=D\left[\frac{1}{s}\left(u+\frac{w}{\tan\alpha }\right)+\mu \frac{\partial u}{\partial s}\right]\\ M_{s}=-K\left[\frac{\partial^{2}w}{\partial s^{2}}+\frac{\mu }{s}\frac{\partial w}{\partial s}\right]\\ M_{\Theta }=-K\left[\frac{1}{s}\frac{\partial w}{\partial s}+\mu \frac{\partial^{2}w}{\partial s^{2}}\right]\\ Q_{s}=\frac{1}{s}\left(M_{s}+s\frac{\partial M_{s}}{\partial s}\right)-\frac{M_{\Theta }}{s} \end{array}$$

with

(27) $$D=\frac{Eh}{1-\mu^{2}},\;K=\frac{Eh^{3}}{12\left(1-\mu^{2}\right)}$$

Substituting Equation (26) into Equation (25) yields

(28) $$\begin{array}{l} D\left(\frac{\partial^{2}u}{\partial s^{2}}+\frac{1}{s}\frac{\partial u}{\partial s}-\frac{u}{s^{2}}+\frac{\mu }{\tan\alpha }\frac{1}{s}\frac{\partial w}{\partial s}-\frac{w}{s^{2}\tan\alpha }\right)+\rho h\omega^{2}u=0\\ K\left(\frac{\partial^{4}w}{\partial s^{4}}+\frac{2}{s}\frac{\partial^{3}w}{\partial s^{3}}-\frac{1}{s^{2}}\frac{\partial^{2}w}{\partial s^{2}}+\frac{1}{s^{3}}\frac{\partial w}{\partial s}\right)+\frac{D}{\tan\alpha }\left(\frac{u}{s^{2}}+\frac{\mu }{s}\frac{\partial u}{\partial s}+\frac{w}{s^{2}\tan\alpha }\right)-\rho h\omega^{2}w=0 \end{array}$$

In order to solve these two ordinary differential equations with variable coefficients, the edge of the shell is divided into *m* segments. In any *j*-th small segment, the coordinate *s* can be taken as the coordinate *s_j_* (*j* = 1, 2, 3, …, *m*) of its midpoint. Then, the coefficients of these two differential equations become constants. The general solution is

(29) $$\begin{array}{l} u=\sum_{i=1}^{6}A_{i}\left(\vartheta_{i}\right)e^{\vartheta_{i}s},\;w=\sum_{i=1}^{6}q_{i}\left(\vartheta_{i}\right)A_{i}\left(\vartheta_{i}\right)e^{\vartheta_{i}s}\\ q_{i}\left(\vartheta_{i}\right)=-\left[\frac{s_{j}^{2}\rho h\omega^{2}}{D}+\left(s_{j}^{2}\vartheta_{i}^{2}+s_{j}\vartheta_{i}-1\right)\right]{\left(\frac{\mu s_{j}\vartheta_{i}-1}{\tan\alpha }\right)}^{-1} \end{array}$$

where $A_{i}\left(\vartheta_{i}\right)$ is an undetermined coefficient. Six roots $\vartheta_{i}$ are obtained by the following equation.

(30) $$\left|\begin{array}{cc} \frac{\mu s_{j}\vartheta -1}{\tan\alpha } & \left(\frac{\rho h\omega^{2}}{D}+\vartheta^{2}\right)s_{j}^{2}+s_{j}\vartheta -1\\ Ks_{j}\vartheta \left(s_{j}^{3}\vartheta^{3}+2s_{j}^{2}\vartheta^{2}-s_{j}\vartheta +1\right)+\frac{Ds_{j}^{2}}{\tan^{2}\alpha }-s_{j}^{4}\rho h\omega^{2} & \frac{\mu s_{j}^{2}\left(1+\mu s_{j}\vartheta \right)}{\tan\alpha } \end{array}\right|=0$$

From Equations (26) and (29), we have

(31) $$U_{j}=P_{j}A_{j}$$

with

(32) $$\begin{array}{l} U_{j}={\left[\begin{array}{cccccc} u & w & \varphi & N_{s} & M_{s} & Q_{s} \end{array}\right]}_{j}^{T}\\ A_{j}={\left[\begin{array}{cccccc} A_{1} & A_{2} & A_{3} & A_{4} & A_{5} & A_{6} \end{array}\right]}_{j}^{T}\\ P_{j}={\left[\begin{array}{cccccc} e^{\vartheta_{1}s} & e^{\vartheta_{2}s} & e^{\vartheta_{3}s} & e^{\vartheta_{4}s} & e^{\vartheta_{5}s} & e^{\vartheta_{6}s}\\ q_{1}e^{\vartheta_{1}s} & q_{2}e^{\vartheta_{2}s} & q_{3}e^{\vartheta_{3}s} & q_{4}e^{\vartheta_{4}s} & q_{5}e^{\vartheta_{5}s} & q_{6}e^{\vartheta_{6}s}\\ q_{1}\vartheta_{1}e^{\vartheta_{1}s} & q_{2}\vartheta_{2}e^{\vartheta_{2}s} & q_{3}\vartheta_{3}e^{\vartheta_{3}s} & q_{4}\vartheta_{4}e^{\vartheta_{4}s} & q_{5}\vartheta_{5}e^{\vartheta_{5}s} & q_{6}\vartheta_{6}e^{\vartheta_{6}s}\\ N_{s1} & N_{s2} & N_{s3} & N_{s4} & N_{s5} & N_{s6}\\ M_{s1} & M_{s2} & M_{s3} & M_{s4} & M_{s5} & M_{s6}\\ Q_{s1} & Q_{s2} & Q_{s3} & Q_{s4} & Q_{s5} & Q_{s6} \end{array}\right]}_{j} \end{array}$$

where T denotes matrix transposition, φ=∂w/∂s.

(33) $$\begin{array}{l} N_{si}=D\left[\vartheta_{i}e^{\vartheta_{i}s}+\frac{\mu }{s}\left(e^{\vartheta_{i}s}+\frac{q_{i}e^{\vartheta_{i}s}}{\tan\alpha }\right)\right]\\ M_{si}=-K\left(q_{i}\vartheta_{i}^{2}e^{\vartheta_{i}s}+\frac{\mu }{s}q_{i}\vartheta_{i}e^{\vartheta_{i}s}\right)\\ Q_{si}=-K\left(q_{i}\vartheta_{i}^{3}e^{\vartheta_{i}s}+\frac{1}{s}q_{i}\vartheta_{i}^{2}e^{\vartheta_{i}s}-\frac{1}{s^{2}}q_{i}\vartheta_{i}e^{\vartheta_{i}s}\right) \end{array}$$

From continuity conditions on the interface between the *j*-th and (*j*+1)-th segments, we have $P_{j+1}\left(jL/m\right)A_{j+1}=P_{j}\left(jL/m\right)A_{j}$. The transfer matrix from the first to the last segment can be obtained as

(34) $$\begin{array}{l} A_{m}=PA_{1}\\ U_{1}\left(0\right)=P_{1}\left(0\right)A_{1},\;\mathrm{at}\;s=0\\ U_{m}\left(L\right)=P_{m}\left(L\right)A_{m},\;\mathrm{at}\;s=L \end{array}$$

where $P=\prod_{j=1}^{m-1}P_{j+1}^{-1}\left(jL/m\right)P_{j}\left(jL/m\right)$. $U_{1}\left(0\right)$ and $U_{m}\left(L\right)$ are the boundary conditions at the top and bottom of the rotating shell, respectively. All undetermined constants can be solved when six boundary conditions are prescribed.

### 3.2. Results and Discussion of Cymbal Disk

As illustrated in Figure 8, the boundary conditions are applied at the bottom, represented by the color yellow: displacement in the *z* direction is set to zero, i.e., *u_z_*(*L*) = 0, and excitation by radial displacement $u_{r}\left(L\right)$. Equation (35) gives the expressions of $u_{r}$ and $u_{z}$. As a free end, the top of the cymbal disk has the boundary conditions: *N*_s_(0) = *M_s_*(0) = *Q_s_*(0). Equation (36) is obtained from the corresponding boundary conditions at the top and bottom of the cymbal disk.

(35) $$\begin{array}{l} u_{r}\left(s\right)=u\left(s\right)\sin\alpha +w\left(s\right)\cos\alpha \\ u_{z}\left(s\right)=-u\left(s\right)\cos\alpha +w\left(s\right)\sin\alpha \end{array}$$

(36) $$\begin{array}{l} U_{1}\left(0\right)={\left[\begin{array}{cccccc} u\left(0\right) & w\left(0\right) & \varphi \left(0\right) & 0 & 0 & 0 \end{array}\right]}^{T}\\ U_{m}\left(L\right)={\left[\begin{array}{cccccc} u\left(L\right) & w\left(L\right) & 0 & N_{s} & M_{s} & Q_{s} \end{array}\right]}^{T} \end{array}$$

Analytical solutions are obtained by the transfer matrix method. The number *m* of segments is taken as 100 to maintain good convergence. The displacement response, *u_z_*(0), at the top of the cymbal disk is obtained under different excitations of radial displacements, *u_r_*(*L*).

**Figure 10:**
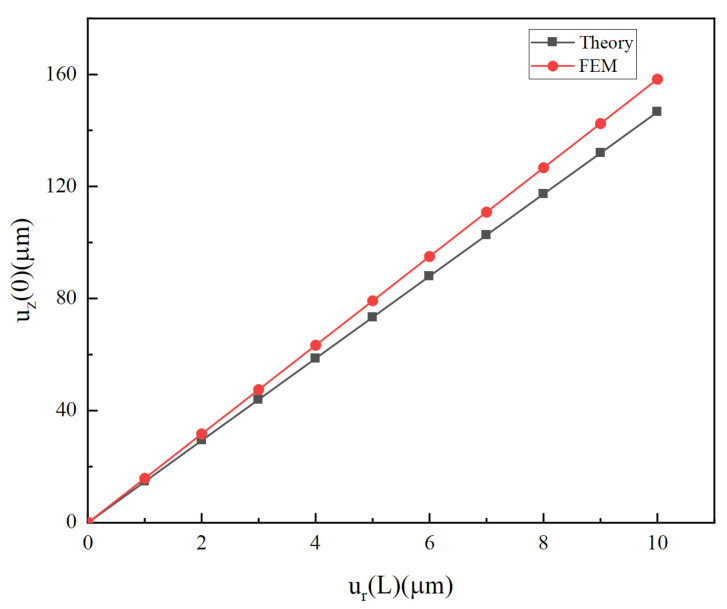
For the cymbal disk, longitudinal displacement on the top versus radial displacement at the bottom.

Static analyses are further carried out by FEM on the Workbench. The mesh is divided by an element size of 0.01 mm to obtain a convergent solution. As a kind of stainless steel, 304SS is selected as a cymbal material because of its easy processing, low cost, and high strength. The boundary condition of the finite element is similar to that of the theory. The results are compared between FEM and theory, as shown in Figure 10. For the cymbal disk, when a radial displacement is applied at the bottom, longitudinal displacement is generated at the top, which is even more than ten times the radial displacement. For example, when the radial displacement at the bottom is 10 μm, a longitudinal displacement of nearly 160 μm is generated on the top, thus the magnification ratio is close to 16. Therefore, the conical shell plays a role in displacement amplification. Finite element results take the average displacement of the top plane as shown in Figure 1, which is slightly larger than theoretical results. The maximum error between the finite element value and the theoretical value is about 8%. The reason is that the influence of the top plane on the displacement is neglected in theoretical analysis.

## 4. Overall Analysis of the Stacked Piezoelectric Cymbal Vibrator

Theoretical and numerical models of stack and cymbal have been studied in the above two sections, respectively. An overall analysis of the stacked piezoelectric cymbal vibrator is further made by considering the coupling between the piezoelectric stack and the cymbal disk.

### 4.1. Coupling between Piezoelectric Stack and Cymbal Disk

At the interface between the piezoelectric stack and cymbal disk, displacements meet the continuity condition.

(37) $$u_{1}\left(r_{1}\right)=u_{r}\left(L\right)$$

where $u_{1}$ and $u_{r}$ represent radial displacements of the electrode layer in the piezoelectric stack and the bottom of the cymbal disk, respectively.

The radial force generated by the upper and lower surfaces of the piezoelectric stack and the force at the bottom of the cymbal disk satisfy the equilibrium equation.

(38) $$n\left(\int_{0}^{h_{1}}T_{r}{}_{r}dz+\int_{h_{1}}^{h_{2}}\sigma_{r2}dz\right)+2F=0$$

where *F* is the radial force with which the piezoelectric stack acts on the bottom of the cymbal.

From Equations (23) and (35), the total longitudinal displacement, *u_T_*, of the structure is written as

(39) $$u_{T}=w_{z}+2u_{z}\left(0\right)$$

where $w_{z}$ is the longitudinal displacement of the piezoelectric stack, and *u_z_*(0) is the longitudinal displacement of the top of the cymbal disk.

### 4.2. Overall Analysis and Comparison

**Figure 11:**
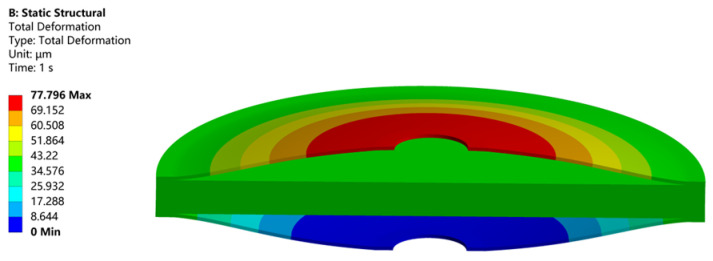
Displacement of the cross section of the overall vibrator.

For the boundary conditions of the overall finite element model of the overall vibrator, the bottom is fixed and the top is free. Furthermore, interfaces between the cymbal and the stack are set as binding contacts. The electrical boundary conditions are illustrated in Figure 2. A static analysis of the overall model is performed. Radial and longitudinal displacements of the overall vibrator under different voltages are obtained. Similarly, harmonic response analysis is also performed to obtain the displacement response of the vibrator at different frequencies. Figure 11 shows the longitudinal displacement response of the vibrator when excitation of voltage 80 V under static analysis. The maximum longitudinal displacement is 77.796 μm.

**Figure 12:**
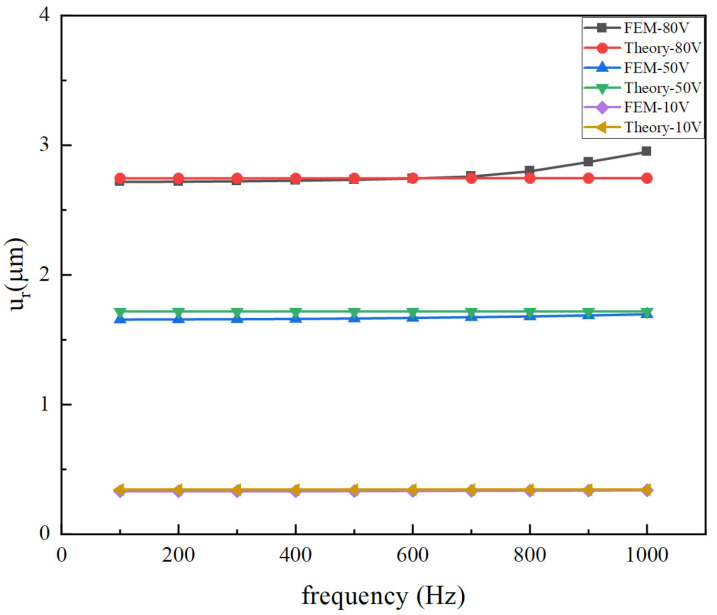
Comparison of radial displacements of the overall vibrator between theory and FEM.

**Figure 13:**
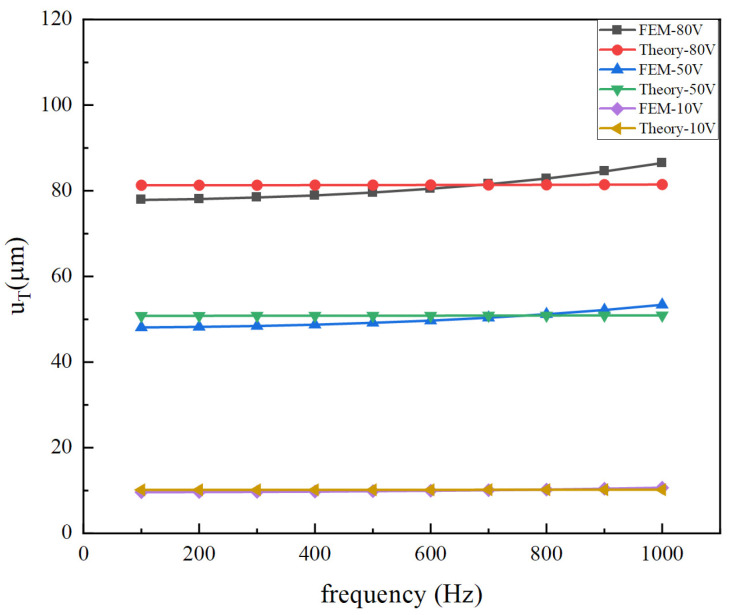
Comparison of total longitudinal displacements of the overall vibrator between theory and FEM for different exciting voltages.

The theoretical model is also calculated and compared with the simulation results. Figure 12 and Figure 13 show the displacement response of the vibrator versus frequency under voltages of 10 V, 50 V, and 80 V, respectively. As can be seen from the figures, the theoretical results are in good agreement with the finite element numerical values. Because the excitation frequency is far less than the natural frequency, the response displacement basically does not change with the increase in the excitation frequency. As a result of the simplification of radial displacement, the result has a large error above 800 Hz. However, at the normal operating frequency (<500 Hz) of the vibrating motor, the theoretical solution is in good agreement with the finite element solution.

There is a remarkable difference between FEM-80 V and Theory-80 V at higher frequencies in the region of 800~1000 Hz. The reason for the difference is that the theoretical model ignores the effect of circumferential displacement, but the finite element model does not. In addition, the overall vibrator has a lower fundamental frequency than that of the piezoelectric stack. Therefore, the driving frequency is closer to the fundamental frequency. Moreover, for a large driving voltage of 80 V, a large circumferential displacement will be generated, and its impact on radial displacement and thickness direction displacement is also greater.

The cymbal disk effectively converts the radial displacement into longitudinal displacement, with a ratio of about 1:30 of radial displacement to longitudinal displacement. It can be noted that the cymbal structure plays an important role in amplifying displacement.

### 4.3. Influence of Cymbal Parameters on Total Longitudinal Displacements of the Overall Vibrator

An analysis is further conducted on the influence of the dimensions and materials of the cymbal disk on the vibration performance of the overall vibrator based on a theoretical model. The harmonic driving voltage with an amplitude of 80 V and a frequency of 100 Hz is adopted.

**Figure 14:**
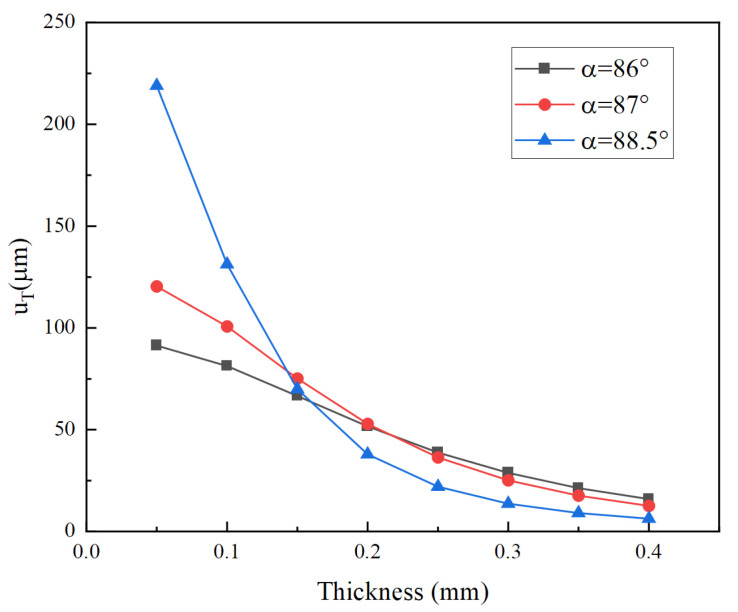
Total longitudinal displacement versus thickness for different angles *α*.

Figure 14 shows the effect of the thickness of the cymbal disks on the total longitudinal displacement of the vibrator at different angles. It can be noted that the thicker the cymbal disk, the smaller the total longitudinal displacement. It is because the stiffness of the cymbal increases with increasing thickness. The deformation then decreases under the same force. Furthermore, for a thin cymbal with a thickness less than 0.2 mm, the larger the inclination angle, the greater the impact of thickness on the total longitudinal displacement.

**Figure 15:**
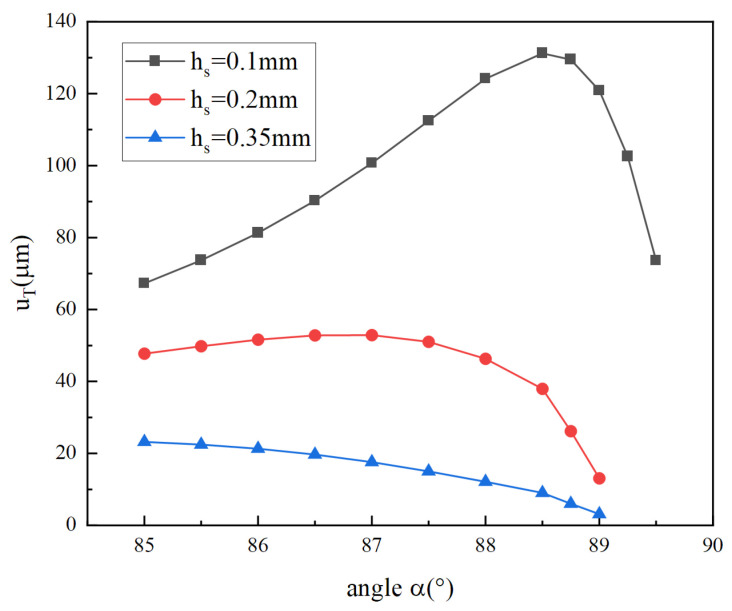
Total longitudinal displacement versus angle α for different thicknesses.

Figure 15 illustrates total longitudinal displacement versus angle α for different thicknesses. The maximum displacement appears at different angles for different thicknesses. The maximum displacement is achieved at *α* = 88.5° for the 0.1 mm-thick cymbal disk. With the increase in thickness, the angle corresponding to maximum displacement decreases gradually. In addition, when the thickness reaches 0.35 mm, the total longitudinal displacement decreases monotonically with the increase in angle in the graph. When the cymbal disk with a fixed thickness is actuated by the piezoelectric stack with the same voltage amplitude and frequency, the longitudinal displacement of the cymbal disk increases with the increase of the coupled radial force and radial displacement. However, as the angle *α* increases, the coupled radial force increases, but the coupled radial displacement decreases. Under these two effects, the maximum longitudinal displacement occurs.

**Figure 16:**
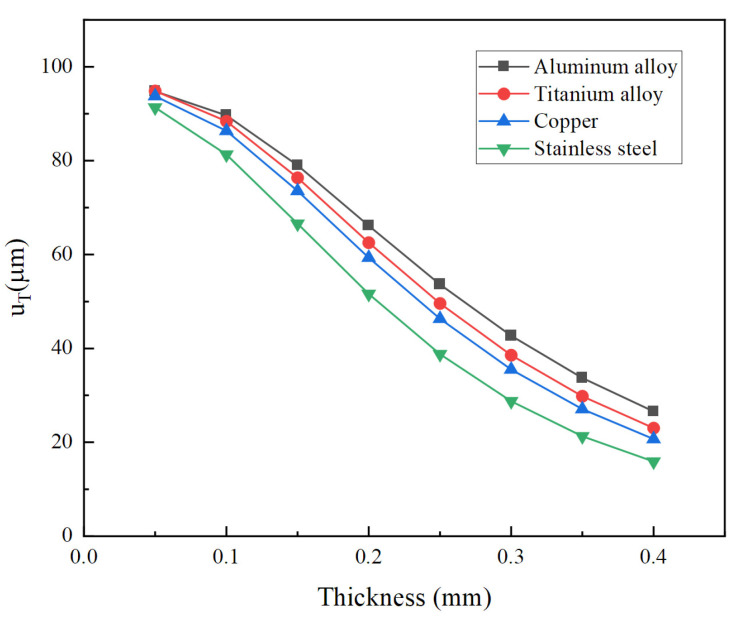
Total longitudinal displacement versus thickness for different materials of cymbal.

In order to investigate the influence of materials on the total longitudinal displacement of the vibrator, several common metal materials are adopted in the calculation. Their material parameters are listed in Table 3. Figure 16 demonstrates total longitudinal displacement versus thickness for different materials of the cymbal disk. For all materials, total longitudinal displacements decrease with an increase in the cymbal disk thickness. Among all materials, aluminum alloy has the largest displacement because it has the smallest Young’s modulus. On the contrary, the smallest displacement is obtained by stainless steel since it has the largest Young’s modulus.

The influence of different factors on the total longitudinal displacement of the vibrator has been investigated. But in practical applications, more factors need to be considered to determine the configuration. For example, a large displacement can be obtained by the thin cymbal. Nevertheless, too thin a cymbal is prone to high stress; Too small an angle would make manufacturing difficult; High-strength materials tend to cost more.

## 5. Conclusions

A theoretical model of the stacked piezoelectric cymbal vibrator was proposed to investigate the coupling between the piezoelectric stack and cymbal disk. The theoretical model overcomes the limitation that the Almen–Laszlo formula cannot calculate the displacement amplification ratio. The theoretical model was calculated by the stress relaxation method and the transfer matrix method. The theoretical model was validated using the finite element method. The influence of the dimensions and materials of the cymbal disk on the vibration performance of the overall vibrator was investigated based on a theoretical model. Amplification ratios of 30 were obtained from radial displacement to longitudinal displacement.

## Figures and Tables

**Table 1:** Parameters of the piezoelectric stack.

Materials	Density *ρ* (kg/m^3^)	Young’s Modulus *E*_2_ (GPa)	Poisson Ratio *μ*_2_	Piezoelectric Constants (C/m^2^)			Thickness (μm)
				*e*_31_	*e*_33_	*e*_15_	
PZT-5H	7500	\	\	−6.5	23.3	17	45
silver	10,490	73	0.38	\	\	\	5

**Table 2:** Material parameters and dimensions of the thin conical shell.

Material	Density *ρ* (kg/m^3^)	Young’s Modulus *E* (GPa)	Poisson Ratio *μ*	*α* (°)	*r*_0_ (mm)	*r*_1_ (mm)	*h* (mm)	*h_S_* (mm)
304SS	7750	193	0.31	86	1.5	7	0.48	0.1

**Table 3:** Material parameters of metallic materials.

Materials	Aluminum Alloy	Titanium Alloy	Copper	Stainless Steel
Density (kg/m^3^)	2770	4620	8960	7750
Young’s modulus (GPa)	71	96	120	193
Poisson ratio	0.33	0.36	0.34	0.31

## Data Availability

Data underlying the results presented in this paper are not publicly available at this time but may be obtained from the authors upon reasonable request.
